# Fibrin-associated diffuse large B-cell lymphoma with plasmacytic differentiation: case report and literature review

**DOI:** 10.1186/s13000-020-01034-7

**Published:** 2020-09-24

**Authors:** Esther Moreno Moreno, Ana Ferrer-Gómez, Héctor Pian Arias, Irene García García, Mónica García-Cosío

**Affiliations:** 1 Department of Pathology, Hospital Universitario Ramón y Cajal, Ctra. de Colmenar Viejo km 9,100, Madrid, 28034 Spain; 2 Department of Haematology, Hospital Universitario Ramón y Cajal, Ctra. de Colmenar Viejo km. 9,100, 28034 Madrid, Spain

**Keywords:** Myxoma, Primary cardiac lymphoma, Fibrin-associated diffuse large B-cell lymphoma, Epstein-Barr virus, Case report

## Abstract

**Background:**

Primary cardiac lymphomas are extremely rare entities (< 2% of cardiac tumours) and the most frequent histologic type is diffuse large B-cell lymphoma (DLBCL). Fibrin-associated DLBCL (FA-DLBCL) is a very unusual form of DLBCL associated with chronic inflammation, and only case reports and small series have been described. In the heart, it usually occurs in the context of a cardiac myxoma or cardiac prostheses and it is not bulk forming. These lymphomas frequently present with non-germinal center phenotype and are associated with Epstein-Barr virus (EBV) type III latency.

**Case presentation:**

We describe a case of FA-DLBCL arising in a cardiac myxoma, with plasmacytic differentiation and type I EBV latency.

**Conclusions:**

Although they are very rare, FA-DLBCLs should be known for their diagnostic difficulty, due to its unspecified clinical manifestations, and for their more favourable prognosis, sometimes even without additional treatment after surgical resection.

## Background

Primary cardiac lymphomas (PCLs) are extremely rare (< 2% of cardiac tumours) [1, 2]. The most frequent histologic type is diffuse large B-cell lymphoma (DLBCL) [1]. Conventional PCLs, such as DLBCL, infiltrate myocardium or pericardium, with aggressive course and low median survival [2]. DLBCL associated with chronic inflammation (DLBCL-CI) occurs in the context of a localized and maintained situation of chronic inflammation and shows association with Epstein-Barr virus (EBV) [3]. The most representative example within this group is pyothorax-associated lymphoma [4]. Fibrin-associated DLBCL (FA-DLBCL) is a very unusual form of this entity, which is mediated by a local immunosuppression mechanism and can be found associated to cardiac myxoma or cardiac prostheses [1].

In FA-DLBCLs, tumour cells are arranged singly or in small clusters within fibrin material [2] or associated to chronic blood effusions [5], with a high proliferative index (Ki-67 > 90%), but without myocardial invasion [1].

The available literature about this entity is scarce and only case reports or small series have been published. These lymphomas usually present with non-germinal center phenotype and associated with EBV type III latency, having better prognosis than conventional DLBCL and DLBCL-CI [2].

We report a case of FA-DLBCL with plasmacytic differentiation and associated to EBV type I latency, arising in a cardiac myxoma and growing in small neoplastic nests. This entity should be know because of its favour outcome, although the diagnosis can be very challenging due to its paucicellular features and, in our case, also to its plasmacytic phenotype with no expression of the panB marker CD20.

## Case presentation

We present a 57-year-old woman, with no significant past medical history, who was hospitalized due to a sudden precordial pain and diagnosed with acute coronary syndrome. Transthoracic echocardiogram revealed a 3 cm-polypoid lesion attached to left atrium. Angioplasty and resection of the lesion were performed.

**Fig. 1:**
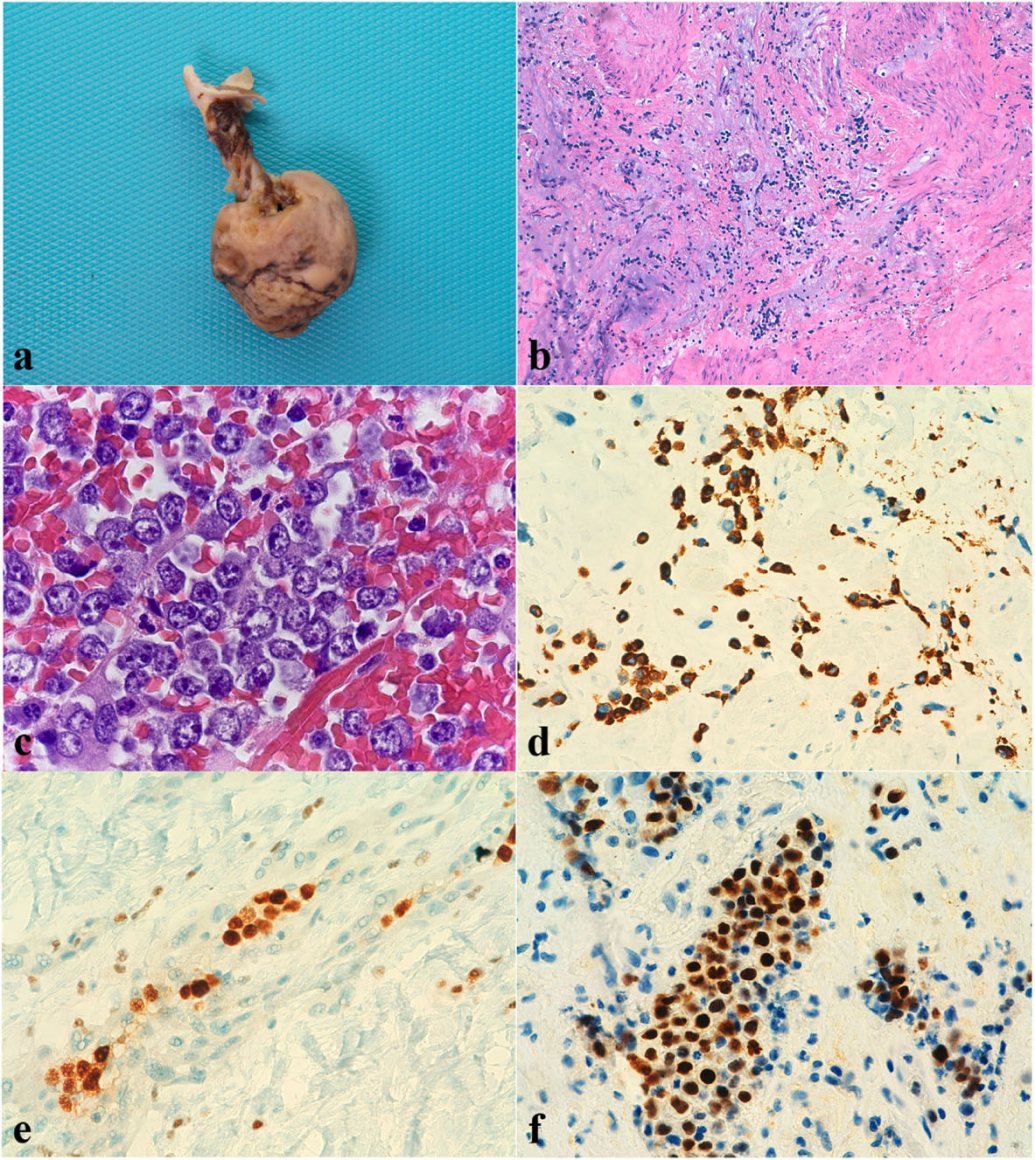
**a** Macroscopic view of 3.5 × 3 cm solid, brownish polypoid specimen with a 2.3 × 0.7 cm pedicle. **b** HE (20x). Histological study revealed a proliferation composed of a dense eosinophilic substance with myxoid basophilic areas, in which spindle and starry cells were observed. No atypia or mitotic figures were identified. **c** HE (100x on oil). Within the myxoma, small aggregates of lymphoid-like cells were identified. High magnification showed vesicular nuclei and conspicuous nucleoli with several mitotic figures and nuclear debris. **d** CD79 (40x). Lymphoma cells were positive for CD79. **e** OCT2 (40x). Lymphoma cells were positive for OCT2. **f** MUM1 (40x). Lymphoma cells showed non-germinal centre phenotype, with MUM1 positivity

**Fig. 2:**
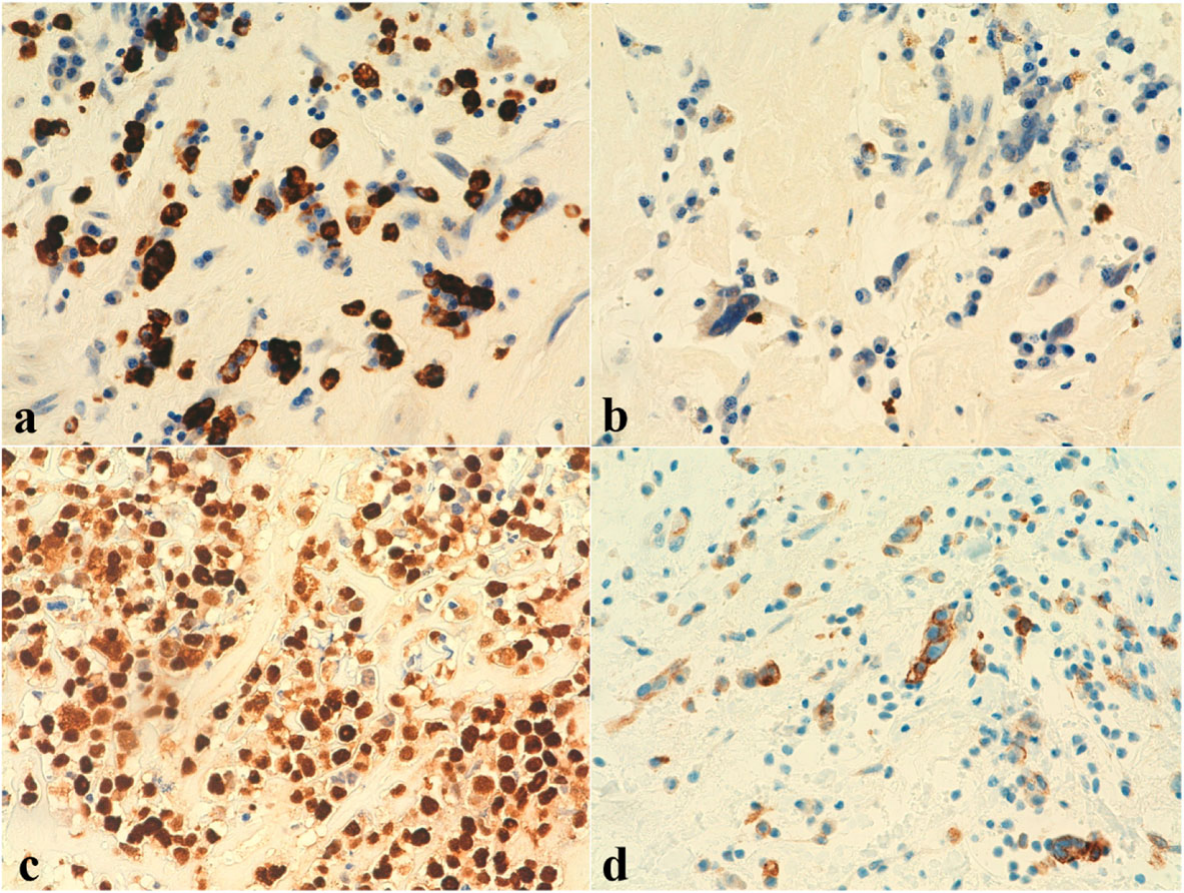
**a** Kappa (HIS 40x) & **b** Lambda (HIS 40x). Malignant cells showed kappa light chain restriction. **c** EBV (40x). EBERs study was positive in malignant cells. **d** PDL1 (40x). Programmed Death-Ligand 1 (PD-L1) study showed positivity in about 60% of the malignant cells

A 3.5 × 3 cm polypoid lesion with a 2.3 × 0.7 cm pedicle was resected. It showed a solid, yellowish, homogeneous cut surface, with a congestive area. Histological study revealed a proliferation composed of a dense eosinophilic substance with myxoid basophilic areas, in which spindle and starry cells were observed. No atypia or mitotic figures were identified. Once immunohistochemical study was performed, these cells showed positivity to Calretinin, CD31, CD34 and Vimentin, confirming the diagnosis of cardiac myxoma. Moreover, within and in the periphery of the myxoma, small aggregates of lymphoid cells were identified. These were large sized cells, with vesicular nuclei and conspicuous nucleoli, showing several mitotic figures and nuclear debris (Fig. 1 a-c). Given these findings, immunohistochemical study was extended and in situ hybridization study for EBV (EBERs) was performed. Such cellularity resulted CD45+, OCT-2+, CD79a+, CD38+, CD20-, PAX5-, MUM1+ and Kappa+, with a proliferation index (Ki67) of 95% (Fig. 1 d-f). Other immunohistochemical markers, such as CD30, CD3, CD138, CD19, Myeloperoxidase, HHV-8, TdT, CD14, CD68 and Lambda were negative. In situ hybridization (HIS) stains for Kappa and Lambda confirmed the kappa light chain restriction. Immunoglobulin gene rearrangement analysis was performed and it resulted clonal. EBERs study was positive and immunohistochemistry for EBV Latent Membrane Protein 1 (LMP1) was negative, with final diagnosis of fibrin-associated DLBCL within a cardiac myxoma, with EBV type I latency, plasmacytic immunophenotype and kappa light chain restriction (Fig. 2).

Programmed Death-Ligand 1 (PD-L1) study showed positivity in about 60% of the malignant cells.

Computed body tomography to staging was not performed, due to the patient was transferred to another hospital one month later.

## Discussion and conclusions

We report an immunocompetent patient who presented with an EBV-DLBCL arising within a cardiac myxoma. She was treated with surgical excision with loss of follow-up 1 month later.

Primary cardiac solid tumours are very unusual entities, in which 77–90% of them are benign, being the most frequent cardiac myxoma [1, 6]. PCL represents 0.5% of extranodal lymphomas [2] and < 2% of primary cardiac tumours. Lymphoproliferative disorders with secondary cardiac involvement are more frequently observed [1]. The most frequent PCL is DLBCL and it is presented as a bulk-forming neoplasm affecting myocardium or pericardium, most frequently in right cavities and clinically manifests as dyspnoea, chest pain due to pericardial effusion, among other symptoms [2]. Conventional PCL has been associated to immunodeficiency and it is diagnosed late in the course of the disease, with an overall survival of 12 months [2].

DLBCL-CI was defined as a new entity in the 2008 World Health Organization (WHO) Classification [3, 4]. These tumours are aggressive DLBCLs, EBV-associated and occur in an enclosed environment with a long-standing chronic inflammation [4], such a pyothorax-associated lymphoma. Few cases of FA-DLBCLs have been described such as case reports and small series (Table 1), usually within cardiac myxoma or associated to cardiac prostheses [7, 8]. Boyer et al. [4] and Svec et al. [9] reported an average age of 55 and 70 years, with a male: female ratio of 2:3 and 1:4, respectively. These tumours are considered a variant of chronic inflammation-associated lymphomas (2017 WHO Classification) [10, 11], with CD20 expression, non-germinal center phenotype, and associated with EBV type III latency, but with better outcomes than the former [2]. FA-DLBCLs occur in immunocompetent patients and appear to be produced by local immunosuppression mechanisms [1]. Characteristically these lymphomas are not bulk forming [2]. Although PCLs are very rare diseases, Gruver et al. [2] reported that fibrin-associated DLBCLs represent 50% of a series of 6 PCLs.

**Table 1:** Summary of clinicopathological features of FA-DLBCL reported cases and our case

Author	Cases	Age	Sex	Location	Diagnosis	Phenotype	EBV latency	Stage	Adjuvant treatment	Follow-up (months)
Garces et al, 2019 [7]	1	50	M	Left atrial myxoma	FA-DLBCL	Non-GC	NA	Localized	None	65-WER
Zanelli et al, 2019 [8]	1	75	F	Adrenal pseudocyst	FA-DLBCL	Non-GC	NA	NA	NA	NA
Boyer et al, 2017 [4]	12	54	F	Left atrial myxoma	FA-DLBCL	NA	NA	NA	NA	130-WER
		55	F	Left atrial myxoma	FA-DLBCL	Non-GC	Type III	NA	None	Died 2 mo-WER
		54	M	Left atrial myxoma	FA-DLBCL	Non-GC	Type III	Localized	None	Died 26 mo-recurrent atrial mass
		56	M	Aortic aneurysm	FA-DLBCL	Non-GC	Type III	Localized	R-CHOP × 6	24-persistent local disease
		68	M	Arterial thromboembolism	FA-DLBCL	Non-GC	Type III	NA	R-COEP × 2	Died 10 mo-WER
		71	M	Aortobifemoral prosthesis	FA-DLBCL	Non-GC	Type II-III	NA	None	10-WER
		79	M	Testicular hematoma	FA-DLBCL	Non-GC	Type III	NA	None	Died 17 mo-WER
		25	M	Subdural hematoma	FA-DLBCL	Non-GC	Type II-III	Localized	None	7-WER
		37	F	Splenic pseudocyst	FA-DLBCL	Non-GC	NA	Localized	R-CHOP × 3	32-WER
		73	M	Retroperitoneal pseudocyst	FA-DLBCL	Non-GC	NA	Localized	R-CHOP × 6	43-WER
		70	M	Adrenal pseudocyst	FA-DLBCL	Non-GC	Type III	Localized	None	14-WER
		44	M	Retroperitoneal pseudocyst	FA-DLBCL	Non-GC	Type III	Localized	CHOP × 5	84-WER
Kirschenbaum et al, 2017 [5]	1	81	M	Arachnoid cyst	FA-DLBCL	NA	NA	Localized	R-L x NA	NA
Yan et al, 2017	4	46	F	Left atrial myxoma	DLBCL-CI	Non-GC	Type III	Localized	None	10-WER
		61	F	Left atrial myxoma	DLBCL-CI	GC	Type III	Localized	None	7-WER
		54	M	Left atrial myxoma	DLBCL-CI	Non-GC	Type III	Localized	None	7-WER
		46	F	Left atrial myxoma	DLBCL-CI	Non-GC	Type III	Localized	None	3-WER
Aguilar et al, 2015 [1]	1	52	M	Left atrial myxoma	FA-DLBCL	Non-GC	Type III	Localized	None	42-WER
Tapan et al, 2015 [6]	1	49	M	Left atrial myxoma	EBV-DLBCL	Non-GC	Type III	Localized	R-CHOP × 6	12-WER
Gruver et a., 2012 [2]	3	55	M	Aortic root graft	FA-DLBCL	Non-GC	Type III	Localized	R-CEOP × 8	16-WER
		56	M	Left atrium	FA-DLBCL	Non-GC	Type III	Localized	R-CHOP × 6	8-WER
		75	M	Mitral valve	FA-DLBCL	Non-GC	EBV -	Localized	R-CVP × 1 and R-CHOP × 6	39-WER
Svec et al, 2012 [4]	1	60	F	Left atrial myxoma	EBV-DLBCL	Non-GC	Type III	Localized	R-CHOP × 6	7-WER
Loong et al, 2010 [3]	4	29	M	Splenic cyst	DLBCL-CI	Non-GC	Type III	Localized	Rituximab × 6	6-WER
		88	M	Right hydrocele	DLBCL-CI	Non-GC	Type III	NA	Lost to follow-up	NA
		70	F	Left atrial myxoma	DLBCL-CI	Non-GC	Type III	Localized	R-CEOP × 4	Died 5 mo-WER
		78	M	Knee prosthesis	DLBCL-CI	Non-GC	Type III	Localized	RT × 7	84 (7 y)-WER
Present case	1	57	F	Left atrial myxoma	Plasmacytic FA-DLBCL	Non-GC	Type I	NA	None	1-Loss of follow-up

*FA-DLBCL* fibrin-associated diffuse large B-cell lymphoma, *DLBCL-CI* DLBCL associated with chronic inflammation, *Non-GC* non-germinal center, *GC* germinal center, *NA* not available, *WER* without evidence of recurrence, *R-L* rituximab, lenalidomide, *R-CHOP* rituximab, cyclophosphamide, doxorubicin, vincristine and prednisone, *R-CVP* rituximab, cyclophosphamide, vincristine and prednisone, *R-COEP* rituximab, cyclophosphamide, vincristine, etoposide, prednisone, *R-CEOP* rituximab, cyclophosphamide, etoposide, oncovin, and prednisone, *RT* radiotherapy

Boyer et al. [4] reported in a 12 cases series that FA-DLBCLs have several differences with DLBCL-CI. DLBCL-CI occurs in older patients, with longer latency and higher mortality. This series is one of the longest series published and this group supports the conclusion that FA-DLBCLs are different entities from DLBCL-CI. FA-DLBCLs arising in cardiac myxomas have lower genetic alterations than DLBCL-CI. Moreover, most FA-DLBCLs represent discrete foci of large B-cells without infiltration of normal tissue and with indolent clinical course [4].

Fibrin-associated DLBCLs are mostly non-germinal center B-cell lymphomas. There is only one case published in the literature diagnosed as lymphoplasmacytic lymphoma, due to its B-cell derivation with loss of CD20 staining and aberrant expression of CD3 [9]. Our case was CD20 negative. Thus, it was diagnosed as fibrin-associated DLBCL, with plasmacytic differentiation.

The association with EBV infection suggests that fibrin-associated DLBCLs are predisposed by an underlying inflammatory background. Type III EBV pattern latency is typically observed in immunosuppressed patients. By contrast, these lymphomas occur in immunocompetent patients, so it is suggested a mechanism of local rather than systemic immunosuppression [1]. This mechanism is not well known, and it could be caused by local expression of proinflammatory cytokines, such as interleukin-6 (IL-6). IL-6 participates in B-cell maturation and several studies have reported that this cytokine is produced in cardiac myxomas. In an enclosed environment, chronic IL-6 exposure might favour the selection B-cells infected by EBV and the evasion of T-cell surveillance [1]. Other cytokines, such as interleukin-10, that inhibits T-cell proliferation, may contribute in local immunosuppression [3].

Despite the frequent association with EBV type III latency in the published cases (22/29 cases, see table), the negativity of LMP-1 in our case suggests a type I EBV latency. In the remaining published cases, EBV latency was not available and one case was EBV negative.

Moreover, it is thought that high levels of PD-L1 expression may contribute to the immune evasion of the EBV-positive B-cells. The study of PD-L1 expression in the reported FA-DLBCLs cases showed a 86% of positivity [4], similarly to our case, that displayed PD-L1 expression in 60% of the neoplastic cells. This may justify in part the type I EBV pattern latency, not associated to immunosuppression, observed in our case.

We present a case of FA-DLBCL with plasmacytic differentiation and type I EBV latency. Although they are very rare, FA-DLBCLs should be known for their diagnostic difficulty, due to its unspecified clinical manifestations and the not bulk forming features with paucity of neoplastic cells. Moreover, in our case the plasmacytic differentiation makes the diagnosis more challenging, because of the lack of expression of panB immunohistochemical markers.

More studies are needed to clarify the pathogenesis of FA-DLBCL, that is probably a different entity from DLBCL-CI in virtue of its excellent outcome, even without adjuvant therapy [1, 2, 4].

## Supplementary information

**Additional file 1.** CARE Checklist.

## Data Availability

´Not applicable´.

## Abbreviations

DLBCL: Diffuse large B-cell lymphoma
FA-DLBCL: Fibrin-associated diffuse large B-cell lymphoma
EBV: Epstein-Barr virus
PCL: Primary cardiac lymphoma
DLBCL-CI: Diffuse large B-cell lymphoma associated with chronic inflammation
EBERs: Hybridization study for Epstein-Barr virus
HIS: In situ hybridization
LMP1: Latent Membrane Protein 1
PD-L1: Programmed Death-Ligand 1
WHO: World Health Organization
